# Distribution of HLA-DRB1 alleles in BRICS countries with a high tuberculosis burden: a systematic review and meta-analysis

**DOI:** 10.1590/0037-8682-0017-2021

**Published:** 2021-07-23

**Authors:** Alice Sarno, Cleidiane Borges Daltro, Carlos Mauricio Cardeal Mendes, Theolis Barbosa

**Affiliations:** 1 Fundação Oswaldo Cruz, Instituto Gonçalo Moniz, Salvador, BA, Brasil.; 2 Universidade Federal da Bahia, Instituto de Ciências da Saúde, Salvador, BA, Brasil.; 3 Rede Brasileira de Pesquisas em Tuberculose, Rio de Janeiro, RJ, Brasil.

**Keywords:** Tuberculosis, Epitope-based vaccine, Rational vaccine design, Vaccine candidate, Major histocompatibility complex, Immunogenicity

## Abstract

**INTRODUCTION:**

Tuberculosis (TB) is the leading cause of death worldwide caused by a single infectious disease agent. Brazil, Russia, India, China, and South Africa (BRICS) account for more than half of the world’s TB cases. Bacillus Calmette-Guérin (BCG) remains the only vaccine available despite its variable efficacy. Promising antigen-based vaccines have been proposed as prophylactic and/or immunotherapeutic approaches to boost BCG vaccination. Relevant antigens must interact with the range of human leukocyte antigen (HLA) molecules present in target populations; yet this information is currently not available.

**METHODS:**

MEDLINE and EMBASE were systematically searched for articles published during 2013-2020 to measure the allelic frequencies of HLA-DRB1 in the BRICS.

**RESULTS:**

In total, 67 articles involving 3,207,861 healthy individuals were included in the meta-analysis. HLA-DRB1 alleles *03, *04, *07, *11, *13, and *15 were consistently identified at high frequencies across the BRICS, with a combined estimated frequency varying from 52% to 80%. HLA-DRB1 alleles *01, *08, *09, *10, *12, and *14 were found to be relevant in only one or two BRICS populations.

**CONCLUSIONS:**

By combining these alleles, it is possible to ensure at least 80% coverage throughout the BRICS populations.

## INTRODUCTION

Tuberculosis (TB), an infectious disease caused by *Mycobacterium tuberculosis*, is the most fatal infectious disease in the world that is caused by a single agent. The emerging countries Brazil, Russia, India, China, and South Africa, all members of a group known as the BRICS, currently account for approximately 50% of all TB cases worldwide as well as 38% of all disease-associated deaths^1^.

The only available vaccine against TB, i.e., BCG, refers in fact to different attenuated strains of *Mycobacterium bovis*, used as part of the immunization schedule in countries and populations where the disease is highly prevalent^2^ ^,^ ^3^. Meta-analyses have shown that BCG offers protection against infection and the development of active TB, when comparing among vaccinated and unvaccinated children^4^. However, the efficacy of BCG varies greatly according to the age and clinical form of TB. While the vaccine is highly protective in children with tuberculous meningitis and miliary TB, its effectiveness varies from 0% to 80% with regard to pulmonary TB^5^, which is responsible for disease transmission, hampering the ability to control disease. 

While the immune response has a role in hampering bacilli multiplication and avoiding disease development, the relative roles of innate and adaptive mechanisms are difficult to weight. Currently, there is a lack of a reliable biomarker to guide the development and evaluation of new vaccine strategies. Nevertheless, progress has been made using strategies that rely on arrays of antigens selected on the basis of inducing powerful Th1 responses^6^ ^,^ ^7^. There is accumulating evidence that points to CD4^+^ T cell involvement and, possibly, the role of these cells in interferon (IFN)-γ production as well as the building of antibody responses as a component of the protective anti-TB immunity^8^ ^-^ ^11^. This is highlighted by the fact that individuals infected with human immunodeficiency virus have a high increase in the risk of developing TB, which is dependent on the grade of deterioration of the CD4^+^ T cell compartment^12^ ^-^ ^13^. 

Antigen presentation to CD4^+^ T lymphocytes triggers the activation and proliferation of these cells, followed by differentiation into effector cytokine-producing cells that migrate to the infected tissue to amplify the bactericidal action of the infected macrophages^14^.

Mycobacterial antigens are presented to CD4^+^ T cells in the context of human leukocyte antigen (HLA) class II molecules. These proteins are encoded by a set of highly polymorphic genes located on the short arm of chromosome 6 and are expressed on the membranes of antigen-presenting cells^15^. Among these genes, HLA-DRB1 alleles are among the most frequently studied. HLA-DRB1 alleles are highly variable, some of which have been associated with susceptibility to active TB disease development. Moreover, the proportions of these alleles can differ greatly among populations^16^. 

New vaccine candidates have been proposed to prevent active disease and transmission, given as a booster to the primary vaccination with BCG. Currently, several candidates are undergoing different phases of clinical trials aimed at protecting newborns and children from infection or protecting adults with latent TB^7^. Among these, promising candidates include epitope-based vaccines that use HLA class II peptide ligands recognized by T cells to generate effective cellular immunity and protection. The impact of HLA class II binding efficiency to relevant epitopes in the immune response against infection at a population level has recently been addressed^17^. To provide high coverage in endemic settings, the present study performed a series of systematic reviews, followed by a meta-analysis, to identify the most relevant HLA alleles for targeting by effective epitopes in the BRICS populations. We could retrieve sufficient literature to describe the proportions of HLA-DRB1 allelic groups in the BRICS countries. 

Systematic reviews and meta-analyses allow for a comprehensive overview of findings from a field of research to avoid bias and to produce a synthesis of comparable studies. Systematic reviews are used to identify studies that are both relevant and of good quality, according to pre-established inclusion, exclusion, and quality grading criteria, while meta-analyses are used to estimate the overall effect or outcome from the findings of the studies selected from a systematic review. Meta-analyses are widely employed in evidence-based medicine to measure the main effect of an intervention or hypothetical causal association of a condition. They are also powerful in achieving the optimized estimate of a given measure across larger numbers of study outcomes, thus reaching broad generalizations that are more robust than those that can be obtained by examining a single study^18^ ^,^ ^19^. Using a systematic review followed by a meta-analysis, we aimed to achieve optimized estimates of HLA-DRB1 allelic frequencies in disease-free individuals in the context of the BRICS, to determine the most relevant alleles to target by epitope-based vaccines.

## METHODS

### Search strategy

Throughout the article search and analysis steps, two investigators (AS and CBD) independently assessed each article, and the results were then compared and validated by consensus. 

A literature search for articles published during 2013-2020 was conducted using two databases: MEDLINE and EMBASE. MEDLINE is the United States National Library of Medicine database, containing more than 24 million references in biomedicine and life sciences from more than 5,000 worldwide journals and books. EMBASE is an Elsevier database, containing more than 29 million references from 8,500 journals, which is focused on the biomedical literature regarding drug, disease, and device information and which includes more than 2,900 peer-reviewed journals not available in MEDLINE. 

For each of the five BRICS countries, the following search terms were used: "HLA [All fields] AND frequency [All fields] AND Brazil", "HLA [All fields] AND frequency [All fields] AND Russia", "HLA [All fields] AND frequency [All fields] AND India", "HLA [All fields] AND frequency [All fields] AND China" and "HLA [All fields] AND frequency [All fields] AND South Africa". Because of the excessive number of articles originating from China retrieved for analysis (2,083 in total), we arbitrarily excluded 50%, that is, only the most recent articles were maintained. Pre-specified inclusion and exclusion criteria were applied in accordance with our study protocol (registered in PROSPERO, CRD # 42018092979).

### Inclusion criteria

The titles and summary sections of the articles retrieved by each search string were accessed, and the following inclusion criteria were considered: articles describing the frequency of HLA-DRB1 alleles (except literature reviews) in healthy individuals from the BRICS, with access provided free of charge, or made available by institutional subscription through the Capes Portal de Periodicos, or by the authors themselves.

### Exclusion criteria

Full-text articles were analyzed, and any studies restricted to patients, i.e., those that did not employ healthy controls, were excluded to avoid the possibility of selection bias, which could correlate the most frequent alleles with the disease studied in the case group. Studies involving individuals from the same family and/or members of tribes, villages, or castes, those that incompletely analyzed the 13 HLA-DRB1 subtypes (HLA-DRB1 *01, *03, *04, *07, *08, *09, *10, *11, *12, *13, *14, *15, and *16), and those reporting only HLA-DQ and/or HLA-DP frequencies were also excluded. The corresponding authors of three studies that analyzed the 13 HLA-DRB1 alleles but did not provide the explicit proportions thereof were contacted by e-mail to request this information. We received one reply from the three authors contacted. This study was maintained in the analysis, and the other two studies were excluded.

### Quality assessment

The methodological quality of the articles was evaluated using a scale adapted from the Newcastle-Ottawa Scale^20^ to preserve applicability in cross-sectional studies ( supplementary material File S1). Only the allelic frequencies of the control groups (healthy individuals) in each study were collected. To be selected for the meta-analysis, studies were required to have a minimum score of 4. 

Article search and selection procedures were performed in accordance with the Preferred Reporting Items for Systematic Reviews and Meta-Analyses (PRISMA) guidelines, as illustrated in File S2 ^21^.

### Data collection

From the articles selected through the systematic review, a database was created to extract data of the relative frequencies for each allele with regard to each BRICS country. In one case, the allelic frequency results were provided after contacting the author by e-mail^22^. All allelic frequencies of HLA-DRB1 were independently collected by two investigators (ASM and CBD). From this database, individual files for each allele and country were generated in comma-separated value format for statistical analyses.

### Statistical analysis

Statistical analyses were performed using the statistical software program R (version 3.3.3, available at https://www.r-project.org/) and the package *meta* (available at https://cran.r-project.org/web/packages/meta/index.html). The average frequencies for individual alleles in each country, as well as the 95% confidence intervals (CIs) and the relative weight of each article, were calculated. The first evaluation used to assess publication bias and heterogeneity among the articles was visual inspection of the funnel plots^23^, followed by Cochran's Q test and the inconsistency measure (I^2^) of the forest plot. Fixed-effect estimates were considered for I^2^ ≤ 50% and p-value >0.05, while random-effect estimates were considered for I^2^ > 50% and p-value <0.05^24^. Forest plots were also used to compare among each of the allelic frequencies of the HLA-DRB1 gene according to each country. 

## RESULTS

### Systematic review

**FIGURE 1:**
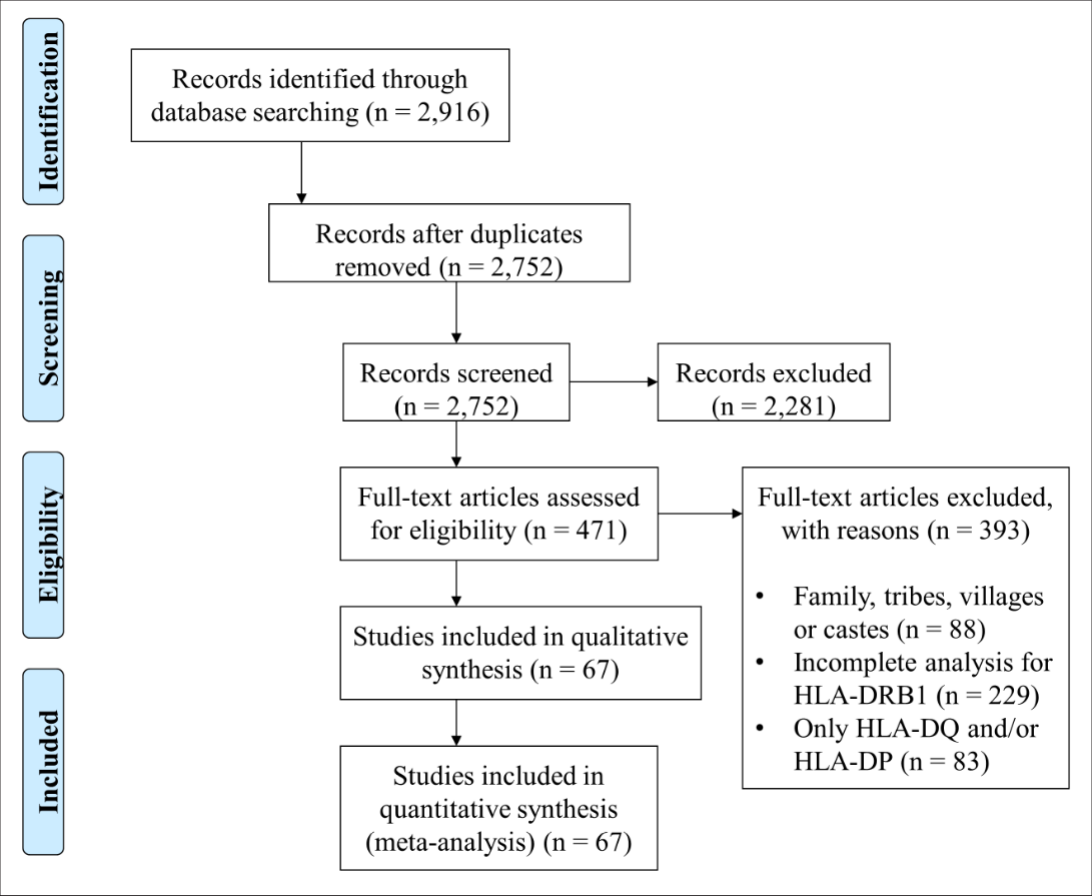
Preferred Reporting Items for Systematic Reviews (PRISMA) flow diagram.

The article search and selection results for all the five search terms are presented in Figure 1. The search and article selection processes for each search term are illustrated in Figures S1-S5.

Overall, during the identification stage, 2,916 articles were analyzed from the five target countries. Of these 2,916 articles, 554 were from Brazil, 188 from Russia, 554 from India, 1,338 from China, and 282 from South Africa. After analyzing the titles and summaries of each article, 394 articles from Brazil, 155 articles from Russia, 336 articles from India, 1,144 articles from China, and 252 articles from South Africa were not included. In these cases, the search either retrieved review articles or, in most cases, articles that presented only the allelic frequencies of HLA class I genes. 

Following the screening stage, 126 articles from Brazil, 33 articles from Russia, 90 articles from India, 192 articles from China, and 30 articles from South Africa were analyzed for eligibility. In total, 393 of these articles were excluded: 104 from Brazil, 27 from Russia, 76 from India, 168 from China, and 18 from South Africa. These excluded studies were performed in restricted populations or families, were restricted to a specific HLA-DRB1 allele, or had presented insufficient data regarding allelic frequencies. 

In the next step, the methodological quality was assessed in the remaining 19 articles from Brazil, six articles from Russia, 14 articles from India, 22 articles from China, and six articles from South Africa, published during 1997-2019. India was the only country in which all the retrieved articles were classified as high quality. Articles from Brazil, Russia, China, and South Africa were classified as of appropriate quality or high quality and were thus considered for meta-analysis. Overall, 39% of the articles included in the meta-analyses were of appropriate quality, while 61% were considered of high quality. 

The inclusion and exclusion criteria were clearly defined in 89% of the articles. Less than half of the studies (46%) did not analyze the full sample and did not specify the rationale for this discrepancy. Overall, the number of samples that were not analyzed was less than 5% of the total samples considered. In 21% of the studies, the frequencies of HLA-DRB1 alleles were not fully presented for all samples. 

The main method used to determine allelic frequencies was single specific primer-polymerase chain reaction (SSP-PCR), which is considered the gold standard method for HLA genotyping^25^. In addition to SSP-PCR, six articles, one from India and five from China, used other typing methods such as sequence-specific oligonucleotide probe-polymerase chain reaction and sequence-based typing-polymerase chain reaction.

Most studies from Brazil were carried out in the South (6) and Southeast (7) regions, with those from the North (1), Northeast (2), and Midwest (1) being less represented. Articles from India were specific to the West (3), North (3), South (5), and Central West (1) regions of the country. Articles from Russia included individuals from the south (1), west (3), and northwest (2) regions, the latter home to two of the most populous cities in this country: Moscow and St. Petersburg, with approximately 12 m and 5 m inhabitants, respectively.

Among the articles published from China, seven were from the eastern region, five were from the south, and six were from the northeast, with fewer articles from the north (2), southeast (2), and central (1) regions. The articles from South Africa encompassed the Central (1), East (2), and Northeast (1) regions of the country. Some articles from Brazil, China, and South Africa included samples from national bone marrow banks, that is, individuals from the entire country, were considered.

Among all articles, 67 were selected for the meta-analysis, corresponding to 3,207,861 healthy individuals from the BRICS countries, distributed as follows: 3,087,960 individuals from Brazil, in 19 articles; 2,333 individuals from Russia in six articles; 3,111 individuals from India in 14 articles; 110,497 healthy individuals from China in 22 articles and 3,960 individuals from South Africa in six articles. The list of articles selected for meta-analysis, with respective locations, methodological quality scores, and sample size, is presented in Table S1.

### Meta-analysis

A meta-analysis was performed to estimate the frequencies of the 13 allelic groups of HLA-DRB1 in populations of BRICS countries. The frequencies, heterogeneity, and p-values obtained for each allele are summarized in Table 1.

**TABLE 1:** Average frequencies of HLA-DRB1 alleles from BRICS populations.

HLA DRB1	COUNTRIES														
	Brazil			Russia			India			China			South Africa		
	PR (%)	CI 95%	I^2^% (p)	PR (%)	CI 95%	I^2^% (p)	PR (%)	CI 95%	I^2^% (p)	PR (%)	CI 95%	I^2^% (p)	PR (%)	CI 95%	I^2^% (p)
*01	**9** ^a^	9-9	7 (0.38)	**11** ^a^	10-13	60 (0.01)	2	1-3	12 (0.3)	2	2-3	97 (0.0001)	7	4-9	89 (0.0001)
*03	**10** ^a^	9-10	46 (0.03)	**9** ^a^	7-11	75 (0.0001)	**9** ^a^	8-10	48 (0.05)	**4ª**	4-5	87 (0.0001)	**21** ^a^	15-27	96 (0.0001)
*04	**11** ^a^	10-13	78 (0.0001)	**11** ^a^	10-12	1 (0.4)	**7** ^a^	5-9	65 (0.04)	**11** ^a^	11-12	84 (0.0001)	**9** ^a^	6-12	93 (0.0001)
*07	**11** ^a^	10-12	70 (0.0001)	**13** ^a^	12-14	0 (0.8)	**14** ^a^	12-16	67 (0.02)	**10** ^a^	9-11	98 (0.0001)	**9** ^a^	7-10	70 (0.005)
*08	**6** ^a^	6-7	59 (0.002)	4	3-4	0 (0.8)	1	0-2	69 (0.01)	**7** ^a^	6-8	91 (0.0001)	2	1-4	91 (0.0001)
*09	1	1-2	81 (0.0001)	1	0-1	58 (0.02)	1	0-1	60 (0.09)	**15** ^a^	14-16	96 (0.0001)	1	1-1	0 (0.8)
*10	2	2-2	0 (0.5)	1	0-1	43 (0.09)	8	6-9	53 (0.03)	1	1-2	61 (0.0001)	2	1-2	64 (0.02)
*11	**13** ^a^	11-14	87 (0.0001)	**13** ^a^	10-16	79 (0.0001)	**7** ^a^	6-10	90 (0.0001)	**6** ^a^	6-6	83 (0.0001)	**15** ^a^	13-18	82 (0.0001)
*12	2	1-2	75 (0.0001)	2	1-2	48 (0.07)	3	2-4	0 (0.5)	12^a^	12-13	86 (0.0001)	3	2-5	88 (0.0001)
*13	**14** ^a^	13-14	39 (0.06)	**11** ^a^	9-13	64 (0.006)	**11** ^a^	10-13	40 (0.1)	**6** ^a^	5-6	90 (0.0001)	**16** ^a^	14-18	65 (0.01)
*14	4	3-5	90 (0.0001)	3	1-4	87 (0.0001)	**9** ^a^	7-11	65 (0.004)	6	6-7	92 (0.0001)	2	0-3	92 (0.0001)
*15	**10** ^a^	9-10	36 (0.08)	**14** ^a^	13-15	0 (0.4)	**23** ^a^	21-24	46 (0.06)	**15** ^a^	14-16	95 (0.0001)	**10** ^a^	9-11	15 (0.3)
*16	4	4-5	55 (0.005)	4	4-5	0 (0.9)	0	0-1	16 (0.3)	2	2-3	96 (0.0001)	0	0-1	52 (0.08)
**CFrq**	84			82			80			86			80		

**PR:** Pooled Results. CFrq, combined estimated frequency for the highlighted alleles.

^a^HLA-DRB1 gene alleles with combined estimated frequencies representative of at least 80% of the populations evaluated. The alleles that are present in relevant proportions in most or all of the BRICS countries are highlighted in grey.

HLA-DRB1 alleles *03, *04, *07, *11, *13, and *15 show combined frequencies varying between 52% and 80% in the BRICS countries. Least variation was observed for HLA-DRB1*04 and *07. Similar frequencies of HLA-DRB1*13 are found in Brazil, Russia, and India, while approximately half of these values are present in China and South Africa. Likewise, HLA-DRB1*11 is present at similar frequencies in Brazil, Russia, and South Africa, but nearly half as much in India and China. The HLA-DRB1*15 allele was found to be present in 23% (95% CI=22-24) of the Indian population, and at least 10% of the populations of the other BRICS countries were considered. The HLA-DRB1*03 allele is most frequent in South Africa, with a frequency of 20% (95% CI=16-25), up to five times higher than that in the other populations studied.

HLA-DRB1 *01, *08, *09, *10, *12, and *14 are relevant in one or two BRICS populations. The HLA-DRB1*01 allele is present at similar frequencies in Brazil, Russia, and South Africa but is present only in approximately 2% of the populations from India and China. The HLA-DRB1*10 allele is present in 9% of the Indian population but is present in less than 2% of other BRICS populations. HLA-DRB1*09 and *12 alleles are present at frequencies of more than 10% in China but less than 5% in the other countries investigated herein. HLA-DRB1*08 and *14 are present in 7% and 6% of the Chinese population, respectively; either of them could be targeted to achieve 80% of minimum coverage in the country. 

The heterogeneity among the studies was generally above 50%. China was the only country wherein heterogeneity was considered significant for all 13 alleles. Accordingly, all allelic frequencies considered for China took into account the random effect estimates. Regarding other countries, the frequencies were partly evaluated by fixed-effects analysis, while the remaining part was analyzed by random-effects analysis. The frequencies of the HLA-DRB1*11 and *14 alleles were estimated using random-effects analysis in all the countries. No heterogeneity was observed for the HLADRB1 alleles *10 in Brazil; *07, *08, *15, and *16 in Russia; *12 in India; and *09 in South Africa.

## DISCUSSION

The present work reports the most frequent HLA-DRB1 alleles in the populations of Brazil, Russia, India, China, and South Africa, which we propose as targets for the development of new vaccines against tuberculosis. We performed a systematic review followed by meta-analysis as methodologies of summarizing the estimates of allelic frequencies, using an adapted scale to judge the quality of the studies retrieved (as there are no previously published scales proposed for this goal). It is important to emphasize that the use of systematic reviews and meta-analyses as a means of reaching broad generalizations beyond the estimates of the effect of specific interventions, although not frequent, has a long-recognized value in a broad range of scientific fields^19^.

We were able to retrieve not less than six articles per country containing data on all the HLA-DRB1 alleles for inclusion in our meta-analysis. The decision to focus only on HLA-DRB1 was supported by our observation, during the identification stage, that the databases contained only 43 articles measuring the frequencies of HLA-DQ or HLA-DP alleles in any of the BRICS countries. Moreover, all these studies reported isolated, specific allelic frequencies, as compared to the ensemble of alleles for these loci. Likewise, an insufficient number of studies have analyzed HLA class I alleles. Liang et al. reported that a significant overlap can occur between HLA class I and class II alleles regarding their specificity to epitopes of the same antigen^17^. However, it is not clear whether a significant overlap is recognized within the same individual.

Given the frequencies found in the aforementioned populations, HLA-DRB1*03, *04, *07, *11, *13, and *15 should be considered as core alleles in the design of new vaccines providing a high coverage throughout the BRICS countries. In addition to these core alleles, HLA-DRB1*01 and *08 in Brazil, HLA-DRB1*01 in Russia, HLA-DRB1*14 in India, and HLA-DRB1*09 and *12 as well as *08 or *14 in China should also be regarded as important targets to yield at least 80% coverage in these specific populations. By contrast, HLA-DRB1*10 and *16 should not be essential targets for new vaccine candidates, as epitopes with low affinity to these alleles, but with high affinity to the remaining discussed alleles, would nonetheless be capable of triggering responses in at least 80% of the BRICS populations.

Among the most frequently found alleles, HLA-DRB1*15, present in at least 10% of all five populations, is associated with a higher incidence of active pulmonary TB and has been considered a possible marker of disease development^26^. Similarly, HLA-DRB1*09, found at a frequency as much as 15 times higher in Chinese populations than in the other BRICS countries studied has also been associated with susceptibility to TB, especially in East Asian populations. Conversely, HLA-DRB1 alleles *03, *07, *12, and *13 are associated with protection against TB as reported in a meta-analysis that examined studies from 12 countries^27^.

Our meta-analysis showed high heterogeneity, which is likely due to the range of study types included^23^ ^,^ ^28^. All studies included herein were considered to be cross-sectional in nature, as we retrieved only results originating from the control groups; however, the original study designs included prospective cohorts and case-control studies. Meta-analyses of cross-sectional studies tend to show high heterogeneity and frequently employ random-effect modeling^28^. Random-effects analysis tends to produce more conservative results, thereby reducing the risk of bias. However, the inclusion of an extensive number of studies is considered to reduce the impact of discrepant observations^29^. Thus, it was possible to account for the differences in sample sizes while still maintaining CIs similar to those calculated using the fixed-effects analysis.

One limitation of this study was the lack of sufficient articles to explore more specific allelic frequencies within all 13 HLA-DRB1 allelic groups. The affinity of the alleles within each allelic group for a given epitope can vary considerably^17^. However, for most HLA-DRB1 allelic groups, one or a few alleles are responsible for a high proportion of their occurrence^17^, and there are tools available to address this issue in vaccine design^30^.

In conclusion, we propose that epitope-based candidates for vaccines against TB should have high affinity to the HLA-DRB1 alleles *03, *04, *07, *11, *13, and *15 as core targets, and to *01, *08, *09, *12, and *14 as additional targets, especially with regard to TB control in the BRICS countries.
