# Supplementary material for: Distribution of HLA-DRB1 alleles in BRICS countries with a high tuberculosis burden: a systematic review and meta-analysis
Source: Rev Soc Bras Med Trop. 2021 Jul 23;54:e0017-2021. doi: 10.1590/0037-8682-0017-2021 (PMC8313104; doi:10.1590/0037-8682-0017-2021)
Supplement: Supplementary file 1 [file 1678-9849-rsbmt-54-e0017-2021-suppl1.pdf]

S1 File. Proposed scale adapted from the Newcastle-Ottawa Scale to cross-sectional studies.

**SAMPLE SELECTION**		***SCORE***
1	*Representability*	
	(_) The study evaluated all individuals from the selected population	1 point
	(_) The samples are representative of the population's mean (probability sampling and measurement by golden-standard)	1 point
	(_) The samples are relatively representative of the population's mean (probability sampling and measurement by golden-standard)	1 point
	(_) The samples were collected from a group of volunteers (no probability sampling and measurement by golden-standard)	0
	(_) No description of the samples' origin	0
2	*The expected results were not previously presented at the beginning of the article*	
	(_) Yes	1 point
	(_) No	0
3	*Randomization of the samples*	
	(_) Yes	1 point
	(_) No	0
	(_) Non-applicable (the study evaluated all the individuals from the selected population (item 1)	1 point
4	*Inclusion and exclusion criteria clearly stated*	
	(_) Yes	1 point
	(_) No	0
**METHODOLOGY**		***SCORE***
1	*Methods for the determination of the variables*	
	(_) The study used two or more methods for the determination of the variables, including the golden-standard method as the principal method (SSP-PCR)	2 points
	(_) The study only used the golden-standard method (SSP-PCR) for the determination of the variables	1 point
	(_) The study didn't use the golden-standard method (SSP-PCR)	0
2	*Aim of the study*	
	(_) The study had as principal aim to evaluate the allele frequencies of the populations	1 point
	(_) The study had as secondary aim to evaluate the allele frequencies of the populations	1 point
**RESULTS**		***SCORE***
1	*Evaluation of the study*	
	(_) The study described an association between variables (allele frequency x population)	1 point
	(_) Results description without any statistical evaluation presented	0
	(_) No description of the results.	0
2	*Losses during sample collection, storage and/or analysis*	
	(_) All collected samples were analyzed	1 point
