# Supplementary figures and images for: Distribution of HLA-DRB1 alleles in BRICS countries with a high tuberculosis burden: a systematic review and meta-analysis
Source: Rev Soc Bras Med Trop. 2021 Jul 23;54:e0017-2021. doi: 10.1590/0037-8682-0017-2021 (PMC8313104; doi:10.1590/0037-8682-0017-2021)

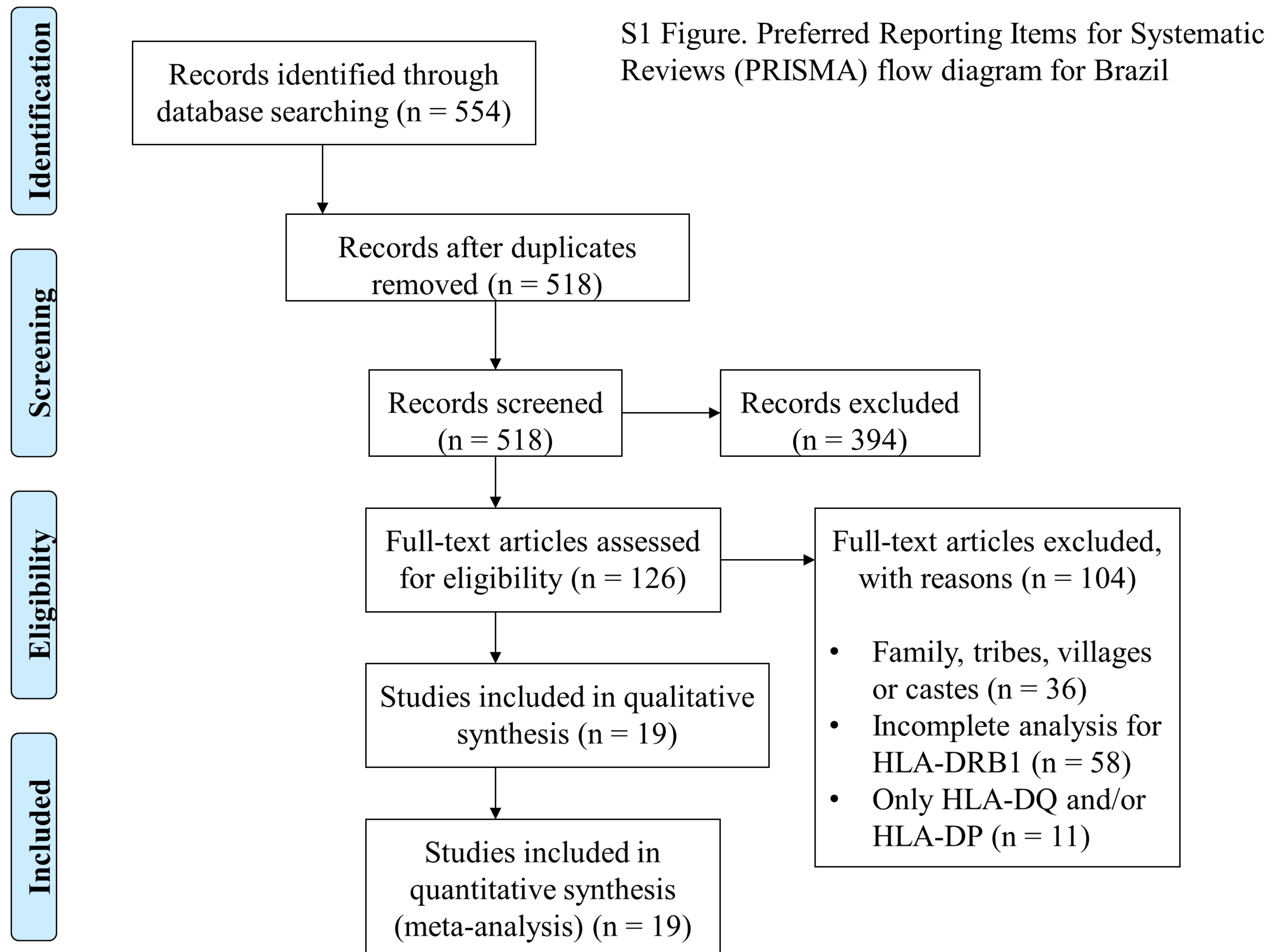

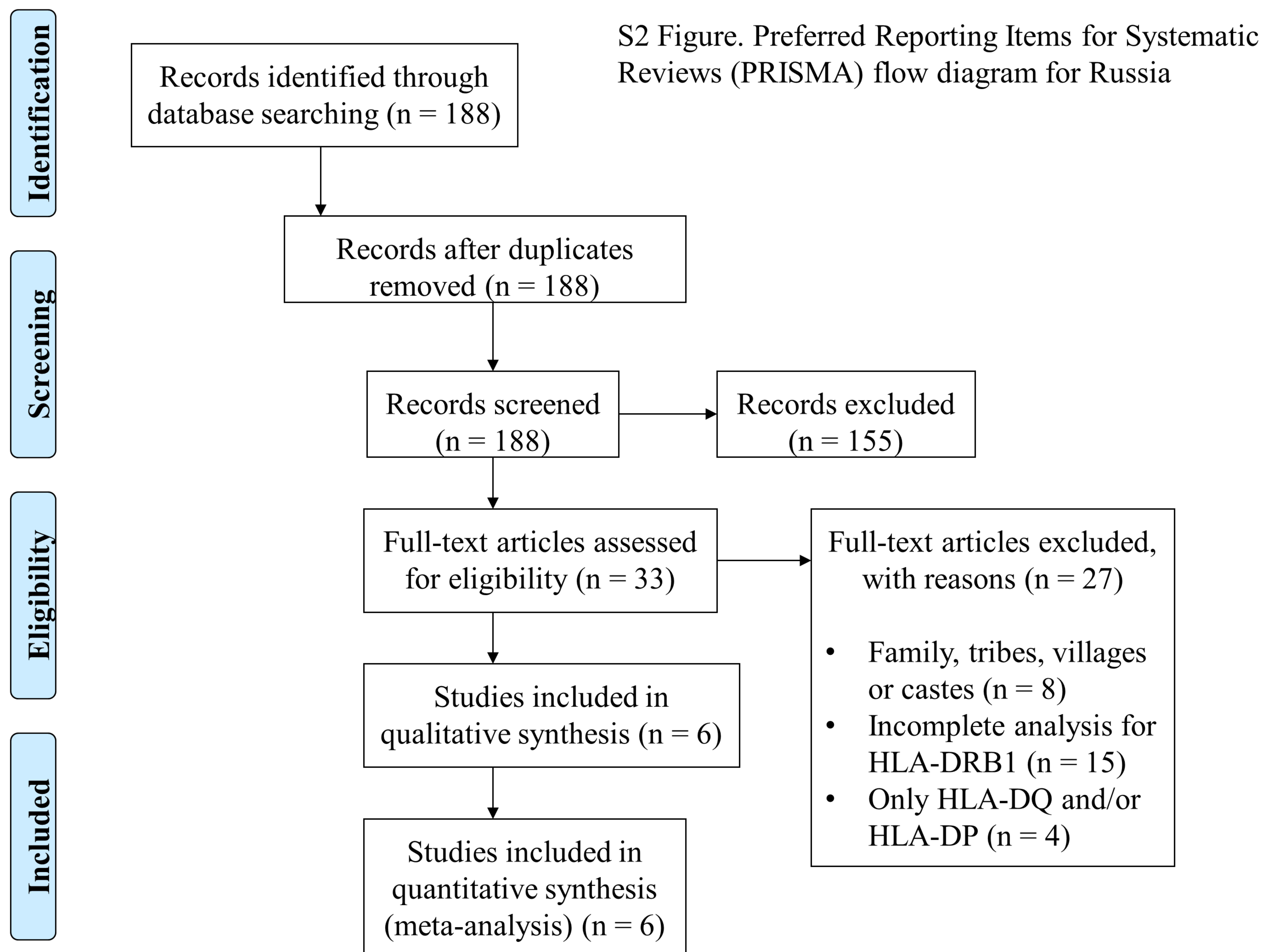

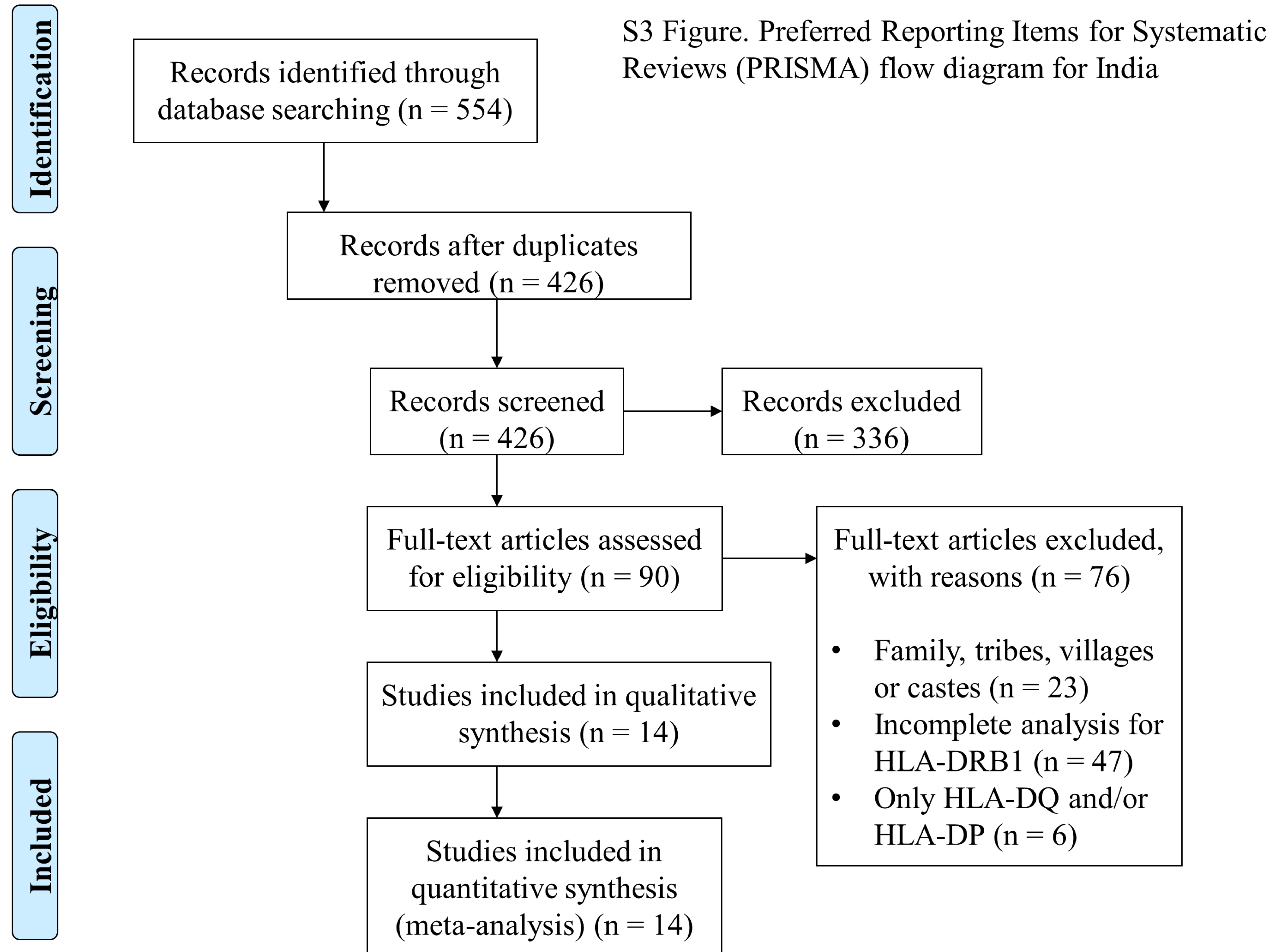

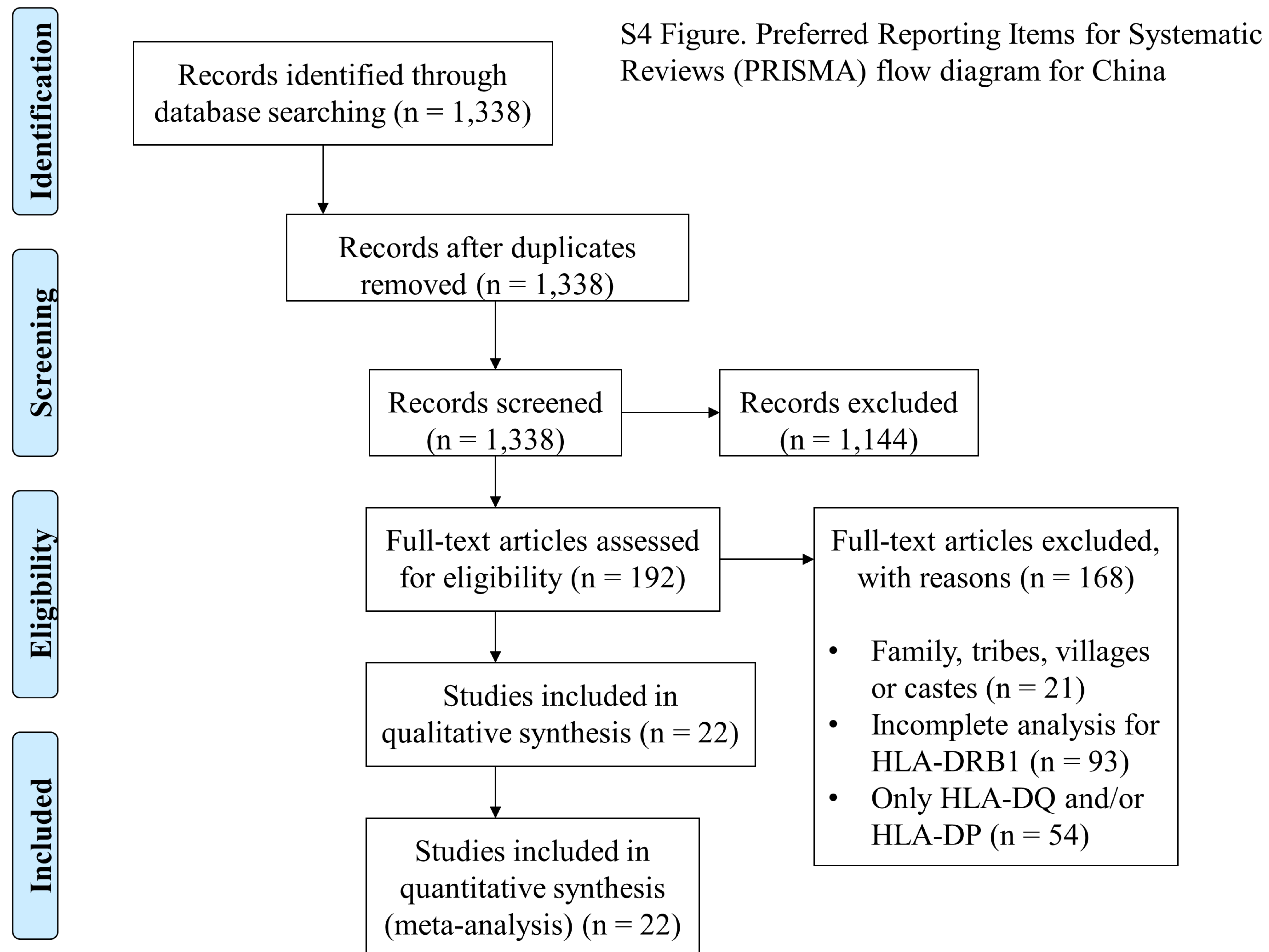

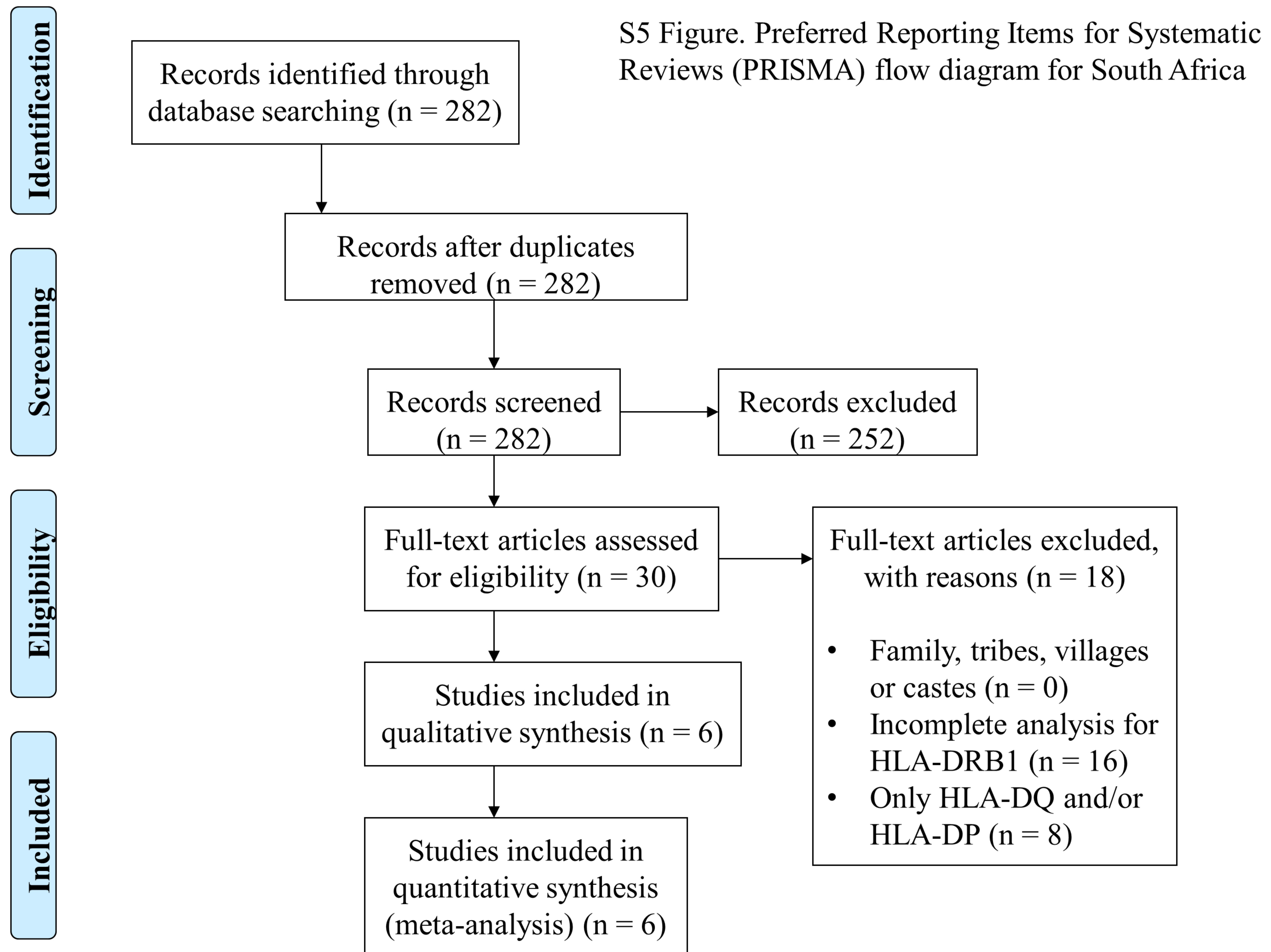

Supplement: Supplementary file 3 [file 1678-9849-rsbmt-54-e0017-2021-suppl3.pdf]
