# Supplementary material for: Distribution of HLA-DRB1 alleles in BRICS countries with a high tuberculosis burden: a systematic review and meta-analysis
Source: Rev Soc Bras Med Trop. 2021 Jul 23;54:e0017-2021. doi: 10.1590/0037-8682-0017-2021 (PMC8313104; doi:10.1590/0037-8682-0017-2021)
Supplement: Supplementary file 4 [file 1678-9849-rsbmt-54-e0017-2021-suppl4.pdf]

S1 Table. Overview of studies included in the systematic reviews and meta-analyses.

STUDIES INCLUDED IN THE SYSTEMATIC REVIEW AND META-ANALYSIS

**First Author**	**Year**	**Location** (City/Region)	**Study design**	**Quality assessment**		***n***
Brazil				Methodological Quality	Score	
Rogel [1]	2019	Sao Paulo/Southeast	Cross-sectional	High	8	120
Torres [2]	2017	Bone Marrow Donor Bank	Cross-sectional	High	8	3,038,286
Caniatti [3]	2017	Maringa/South	Cross-sectional	High	7	147
Barbosa [4]	2016	Sao Paulo/Southeast	Cross-sectional	High	8	112
Ferreira [5]	2014	Unspecified/North	Case-control	Appropriate	6	506
Baleotti [6]	2014	Marilia/Southeast	Case-control	Appropriate	6	2,016
Carvalho [7]	2013	Teresina/Northeast	Cross-sectional	High	9	43,878
Ribas-Silva [8]	2013	Maringa/South	Case-control	Appropriate	6	520
Corrêa [9]	2012	São Luis/Northeast	Case-control	Appropriate	6	170
Gonzaga [10]	2013	Marilia/Southeast	Case-control	High	7	1,052
Souza [11]	2012	Maringa/South	Case-control	Appropriate	6	408
Usnayo [12]	2011	Rio de Janeiro/Southeast	Case-control	Appropriate	5	432
da Silva [13]	2009	Maringa/South	Case-control	High	8	434
Rassi [14]	2006	Unspecified	Case-control	High	7	240
Cardoso [15]	2005	Campinas/South	Case-control	High	8	190
Pavoni [16]	2003	Campo Grande/Midwest and Curitiba/South	Case-control	High	7	769
Freitas [17]	2004	Ribeirao Preto/Southeast	Case-control	High	8	333
Fernandes [18]	2002	Ribeirao Preto/Southeast	Case-control	High	8	361
Total						3,087,960
Russia						
Artem’eva [19]	2013	Moscow/West	Cross-sectional	High	7	878
Smagina [20]	2012	Barnaul/South	Case-control	Appropriate	6	200
Golovanova [21]	2009	West Siberia/West	Cross-sectional	Appropriate	6	210
Favorova [22]	2006	Moscow/West	Case-control	Appropriate	6	447

S1 Table (continued). Overview of studies included in the systematic reviews and meta-analyses.

STUDIES INCLUDED IN THE SYSTEMATIC REVIEW AND META-ANALYSIS						
**First Author**	**Year**	**Location** (City/Region)	**Study design**	**Quality assessment**		***n***
Russia				Methodological Quality	Score	
Kapustin [23]	2001	St. Petersburg/ Northwest	Case-control	High	7	200
Kapustin [24]	1997	St. Petersburg/ Northwest	Case-control	Appropriate	6	398
Total						2,333
India						
Katkam [25]	2018	Hyderabad/South	Case-control	High	7	227
Ramgopal [26]	2018	Unspecified/South	Case-control	High	7	235
Konda Mohan [27]	2017	Chennai/East	Case-control	High	7	100
Aggarwal [28]	2016	Unspecified	Case-control	High	7	54
Rathika [29]	2016	Tamil Nader/South	Case-control	High	7	47
Prasannavar [30]	2014	Mumbai/West	Case-control	High	7	180
Patel [31]	2013	Unspecified/Central-West	Case-control	High	7	550
Negi [32]	2012	Chandigarh/North	Case-control	High	8	199
Chhaya [33]	2010	Mumbai/West	Cross-sectional	High	9	170
Tiercy [34]	2010	Madurai/South	Case-control	High	7	224
Agrawal [35]	2007	Lucknow/North	Cross-sectional	High	7	404
Selvaraj [36]	2007	Chennai/South	Case-control	High	7	116
Gulati [37]	2007	Lucknow/North	Case-control	High	7	404
Amarapurpar [38]	2003	Mumbai/West	Case-control	High	8	201
Total						3,111
China						
Wang [39]	2019	Xinjang Uygur Autonomous Region/Northwest	Case-control	High	7	123
Hu [40]	2017	Unspecified	Case-control	Appropriate	6	130
Shao [41]	2016	Dalian/North	Cross-sectional	Appropriate	6	29,058
Ding [42]	2016	Shandong/East	Case-control	High	7	501
Liu [43]	2015	Heilongjiang/Northeast	Case-control	High	7	66
Wang [44]	2014	Hangzhou/East	Cross-sectional	Appropriate	6	12,768

S1 Table (continued). Overview of studies included in the systematic reviews and meta-analyses.

STUDIES INCLUDED IN THE SYSTEMATIC REVIEW AND META-ANALYSIS						
**First Author**	**Year**	**Location** (City/Region)	**Study design**	**Quality assessment**		***n***
China				Methodological Quality	Score	
He [45]	2014	Shanghai/South; Hebei/North; Sichuan/Southwest; Hunan/South-Central	Case-control	High	7	916
Li [46]	2014	Unspecified/Northeast	Cross-sectional	High	7	40,929
Zhao [47]	2013	Unspecified/East	Case-control	High	8	2,027
Liu [48]	2012	Guangzhou/South	Case-control	High	8	416
He [49]	2012	Bone Marrow Donor Bank	Case-control	High	8	6,591
Li [50]	2012	Harbin/Northeast	Case-control	High	7	410
Sun [51]	2012	Guangzhou/South	Case-control	Appropriate	6	1,434
Qin [52]	2011	Jiangsu/ East	Cross-sectional	Appropriate	6	6,476
Wang [53]	2011	Guangzhou/South	Case-control	Appropriate	6	174
Jiang [54]	2011	Unspecified/East	Case-control	High	7	1,000
Xie [55]	2011	Beijing/Northeast	Case-control	Appropriate	6	320
Lu [56]	2010	Hefei/East	Case-control	Appropriate	6	508
Shen [57]	2010	Guanzhong/Northeast	Cross-sectional	High	7	20,000
Wu [58]	2009	Jiangxi, Hunan and Fujian/South	Case-control	High	8	182
Hei [59]	2009	Beijing/Northeast; Suzhou/East; Shenzhen/South	Cross-sectional	Appropriate	6	1,436
Zhang [60]	2009	Jinan/East	Case-control	Appropriate	6	1,054
Huang [61]	2009	Chongqing/Southwest	Case-control	Appropriate	6	4,957
Total						110,497
South Africa						
Paximadis [62]	2012	Unspecified	Cross-sectional	High	8	568
Lombard [63]	2006	Venda/Northeast	Case-control	Appropriate	6	234
Bhimma [64]	2002	Durban/East	Case-control	Appropriate	5	897
Patel [65]	2002	Johannesburg/Central	Case-control	Appropriate	6	166
Pirie [66]	2001	Durban/East	Case-control	Appropriate	4	1,180
Schipper [67]	1997	Bone Marrow Donor Bank	Cross-sectional	Appropriate	6	915
Total						3,960

References

[1] Rogel CS, De Souza-Santana FC, Marcos EVC, Ogawa MM, Basso G, Tomimori J. HLA alleles in renal transplant recipients with nonmelanoma skin cancer in southeastern Brazil. An Bras Dermatol 2019;94:287–92. doi:10.1590/abd1806-4841.20197322.

[2] Torres L, da Silva Bouzas LF, Almada A, de Sobrino Porto LCM, Abdelhay E. Distribution of HLA-A, -B and -DRB1 antigenic groups and haplotypes from the Brazilian bone marrow donor registry (REDOME). Hum Immunol 2017;78:602–9. doi:10.1016/j.humimm.2017.08.002.

[3] da Costa Lima Caniatti MC, Borelli SD, Guilherme ALF, Tsuneto LT. Association between HLA genes and dust mite sensitivity in a Brazilian population. Hum Immunol 2017;78:88–94. doi:10.1016/j.humimm.2016.10.014.

[4] Barbosa ÂM, Prestes-Carneiro LE, Sobral ARS, Sakiyama MJ, Lemos BC, Abreu MAMM de, et al. Lack of association between alopecia areata and HLA class I and II in a southeastern Brazilian population. An Bras Dermatol 2016;91:284–9. doi:10.1590/abd1806-4841.20164250.

[5] Ferreira AR, Singh B, Cabrera-Mora M, Magri De Souza AC, Queiroz Marques MT, Porto LCS, et al. Evaluation of Naturally Acquired IgG Antibodies to a Chimeric and Non-Chimeric Recombinant Species of Plasmodium vivax Reticulocyte Binding Protein-1: Lack of Association with HLA-DRB1*/DQB1* in Malaria Exposed Individuals from the Brazilian Amazon. PLoS One 2014;9:e105828. doi:10.1371/journal.pone.0105828.

[6] Baleotti W, Ruiz MO, Fabron A, Castilho L, Giuliatti S, Donadi EA. HLA-DRB1 *07:01 allele is primarily associated with the Diego a alloimmunization in a Brazilian population. Transfusion 2014;54:2468–76. doi:10.1111/trf.12652.

[7] Carvalho MG, Tsuneto LT, Moita Neto JM, Sousa LCDM, Sales Filho HLA, Macêdo MB, et al. HLA-A, HLA-B and HLA-DRB1 haplotype frequencies in Piauí's volunteer bone marrow donors enrolled at the Brazilian registry. Hum Immunol 2013;74:1598–602. doi:10.1016/J.HUMIMM.2013.08.283.

[8] Ribas-Silva RC, Ribas AD, Dos Santos MCG, da Silva W V, Lonardoni MVC, Borelli SD, et al. Association between HLA genes and American cutaneous leishmaniasis in endemic regions of Southern Brazil. BMC Infect Dis 2013;13:198. doi:10.1186/1471-2334-13-198.

[9] Corrêa R da GCF, Aquino DMC de, Caldas A de JM, Serra H de O, Silva FF, Ferreira M de JC, et al. Association analysis of human leukocyte antigen class II (DRB1) alleles with leprosy in individuals from São Luís, state of Maranhão, Brazil. Mem Inst Oswaldo Cruz 2012;107:150–5. doi:10.1590/S0074-02762012000900022.

[10] Gonzaga HFS, Marcos EVC, Santana FCS, Jorge MA, Tomimori J. HLA alleles in Brazilian patients with fissured tongue. J Eur Acad Dermatology Venereol 2013;27:e166–70. doi:10.1111/j.1468-3083.2012.04537.x.

[11] Souza CF, Noguti EN, Visentainer JEL, Cardoso RF, Petzl-Erler ML, Tsuneto LT. HLA and MICA genes in patients with tuberculosis in Brazil. Tissue Antigens 2012;79:58–63. doi:10.1111/j.1399-0039.2011.01789.x.

[12] Usnayo MJG, Andrade LEC, Alarcon RT, Oliveira JC, Silva GMF, Bendet I, et al. Estudo da frequência dos alelos de HLA-DRB1 em pacientes brasileiros com artrite reumatoide. Rev Bras Reumatol 2011;51:474–83. doi:10.1590/S0482-50042011000500007.

[13] da Silva SA, Mazini PS, Reis PG, Sell AM, Tsuneto LT, Peixoto PR, et al. HLA-DR and HLA-DQ alleles in patients from the south of Brazil: markers for leprosy susceptibility and resistance. BMC Infect Dis 2009;9:134. doi:10.1186/1471-2334-9-134.

[14] Rassi DM, Wastowski IJ, Simoes RT, Rodrigues S, Deghaide NNH., Mendes-Junior CT, et al. TNFa-e Microsatellite, HLA-DRB1 and -DQB1 Alleles and Haplotypes in Brazilian Patients Presenting Recently Diagnosed Type 1 Diabetes Mellitus. Ann N Y Acad Sci 2006;1079:300–4. doi:10.1196/annals.1375.045.

[15] Cardoso CB, Uthida-Tanaka AM, Magalhães RF, Magna LA, Kraemer MHS. Association between psoriasis vulgaris and MHC-DRB, -DQB genes as a contribution to disease diagnosis. Eur J Dermatol 2005;15:159–63.

[16] Pavoni DP, Roxo VMMS, Marquart Filho A, Petzl-Erler ML. Dissecting the associations of endemic Pemphigus Foliaceus (Fogo Selvagem) with HLA-DRB1 alleles and genotypes. Genes Immun 2003;4:110–6. doi:10.1038/sj.gene.6363939.

[17] Freitas M, Silva D, Deghaide N, Donadi E, Louzada-Júnior P. Is HLA class II susceptibility to primary antiphospholipid syndrome different from susceptibility to secondary antiphospholipid syndrome? Lupus 2004:125–31. doi:10.1191/0961203304lu520oa.

[18] Fernandes APM, Louzada-Junior P, Foss MC, Donadi EA. HLA-DRB1, DQB1, and DQA1 Allele Profile in Brazilian Patients with Type 1 Diabetes Mellitus. Ann N Y Acad Sci 2006;958:305–8. doi:10.1111/j.1749-6632.2002.tb02992.x.

[19] Artem'eva O V., Kostomarova I V., Serova LD. The clinical and genetic characteristics of long-lived people from the Moscow region. Adv Gerontol 2014;4:95–101. doi:10.1134/S2079057014020039.

[20] Smagina I V., Elchaninova SA, Zolovkina AG, Ignatova YN, Kudryavtseva EA. Genetic Risk Factors for Multiple Sclerosis in the Population of the Altai District. Neurosci Behav Physiol 2012;42:876–9. doi:10.1007/s11055-012-9652-5.

[21] Golovanova O V, Konenkov VI, Shevchenko A V, Smol'nikova M V. Frequencies of the DRB1, DQA1, DQB1 and TNFA alleles in

immigrant population of West Siberia. Genetika 2009;45:1118–24.

[22] Favorova OO, Favorov A V, Boiko AN, Andreewski T V, Sudomoina MA, Alekseenkov AD, et al. Three allele combinations associated with multiple sclerosis. BMC Med Genet 2006;7:1–9. doi:10.1186/1471-2350-7-63.

[23] Kapustin SI, Popova TI, Lyshchov AA, Imyanitov EN, Blinov MN, Abdulkadyrov KM. HLA-DR4-Ala74 beta is associated with risk and poor outcome of severe aplastic anemia. Ann Hematol 2001;80:66–71.

[24] Kapustin SI, Popova TI, Lyschov AA, Togo A V, Abdulkadyrov KM, Blinov MN. HLA-DR2 Frequency Increase in Severe Aplastic Anemia Patients is Mainly Attributed to the Prevalence of DR15 Subtype. Pathol Oncol Res 1997;3:106–8.

[25] Katkam SK, Rajasekhar L, Kutala VK. The influence of functional polymorphic positions of HLA-DRβ1 molecules on risk for South Indian systemic lupus erythematosus patients. Lupus 2018;27:991–1000. doi:10.1177/0961203318759200.

[26] Ramgopal S, Rathika C, Padma MR, Murali V, Arun K, Kamaludeen MN, et al. Interaction of HLA-DRB1* alleles and CTLA4 (+ 49 AG) gene polymorphism in Autoimmune Thyroid Disease. Gene 2018;642:430–8. doi:10.1016/j.gene.2017.11.057.

[27] Konda Mohan V, Ganesan N, Gopalakrishnan R, Venkatesan V. HLA-DRB1 shared epitope alleles in patients with rheumatoid arthritis: relation to autoantibodies and disease severity in a south Indian population. Int J Rheum Dis 2017;20:1492–8. doi:10.1111/1756-185X.12948.

[28] Aggarwal R, Gupta A, Naru J, Berka N, Nanda N, Suri D, et al. HLA-DRB1 in Henoch-Schönlein purpura: A susceptibility study from North India. Hum Immunol 2016;77:555–8. doi:10.1016/j.humimm.2016.05.009.

[29] Rathika C, Murali V, Dhivakar M, Kamaraj R, Malini RP, Ramgopal S, et al. Susceptible and protective associations of HLA alleles and haplotypes with cervical cancer in South India. Asian Pacific J Cancer Prev 2016;17:2491–7. doi:10.7314/APJCP.2016.17.5.2491.

[30] Prasannavar DJ, Yeola A, Pradhan V, Patwardhan M, Rajadhyaksha A, Ghosh K. Distribution of HLA-DRβ1 alleles among well-characterized rheumatoid arthritis patients from Western India. Rheumatol Int 2014;34:705–8. doi:10.1007/s00296-013-2758-4.

[31] Patel JS, Patel MM, Koringa PG, Shah TM, Patel AK, Tripathi AK, et al. Human leukocyte antigen alleles, genotypes and haplotypes frequencies in renal transplant donors and recipients from West Central India. Indian J Hum Genet 2013;19:219–32. doi:10.4103/0971-6866.116122.

[32] Negi RR, Bhoria P, Pahuja A, Saikia B, Varma N, Malhotra P, et al. Investigation of the Possible Association between the HLA Antigens

and Idiopathic Thrombocytopenic Purpura (ITP). Immunol Invest 2012;41:117–28. doi:10.3109/08820139.2011.593218.

[33] Chhaya S, Desai S, Saranath D. HLA polymorphisms in Sindhi community in Mumbai, India. Int J Immunogenet 2010;37:373–7. doi:10.1111/j.1744-313X.2010.00936.x.

[34] Tiercy J-M, Rathinam SR, Gex-Fabry M, Baglivo E. A shared HLA-DRB1 epitope in the DR beta first domain is associated with Vogt-Koyanagi-Harada syndrome in Indian patients. Mol Vis 2010;16:353–8.

[35] Agrawal S, Khan F, Bharadwaj U. Human genetic variation studies and HLA class II loci. Int J Immunogenet 2007;34:247–52. doi:10.1111/j.1744-313X.2007.00683.x.

[36] Selvaraj P, Nisha Rajeswari D, Jawahar M, Narayanan P. Influence of HLA-DRB1 alleles on Th1 and Th2 cytokine response to Mycobacterium tuberculosis antigens in pulmonary tuberculosis. Tuberculosis 2007;87:544–50. doi:10.1016/j.tube.2007.08.001.

[37] Gulati S, Tripathi P, Patil SJ, Sharma RK, Agarwal S. Is typing for HLA class II alleles beneficial in Indian children with idiopathic nephrotic syndrome? Pediatr Nephrol 2007;22:528–32. doi:10.1007/s00467-006-0365-2.

[38] Amarapurpar D, Patel N, Kankonkar S, Resident S. HLA Class II Genotyping in Chronic Hepatitis B Infection 2003;51.

[39] Wang L, Li B, Tie X, Liu T, Zheng S, Liu Y. Association between *HLA-DRB1* * allele polymorphism and caries susceptibility in Han Chinese children and adolescents in the Xinjiang Uygur Autonomous Region. J Int Med Res 2019:030006051989385. doi:10.1177/0300060519893852.

[40] Hu Y, Wu J-Z, Zhu H, Zhang S-H, Zhu Y-Y, Wu Y-Y, et al. Association of HLA-DRB1, HLA-DQB1 Polymorphisms with HPV 16 E6 Variants among Young Cervical Cancer Patients in China. J Cancer 2017;8:2401–9. doi:10.7150/jca.19809.

[41] Shao LN, Zhang ST, Yu WJ, Zhou SH, Duan Y, Pan LZ, et al. HLA-A, HLA-B, HLA-DRB1 allele and haplotype frequencies of 14 529 Chinese Han bone marrow donors living in Dalian, China. Int J Immunogenet 2016;43:79–85. doi:10.1111/iji.12248.

[42] Ding S, Zhang Y, Zhang X, Jiang X, Pang B, Song Y, et al. Correlation Between HLA-A, B and DRB1 Alleles and Severe Fever with Thrombocytopenia Syndrome 2016. doi:10.1371/journal.pntd.0005076.

[43] Liu GL, Cao FL, Zhao MY, Shi J, Liu SH. Associations between HLA-A\B\DRB1 polymorphisms and risks of vulvar lichen sclerosus or squamous cell hyperplasia of the vulva. Genet Mol Res 2015;14:15962–71. doi:10.4238/2015.December.7.8.

[44] Wang F, He J, Chen S, Qin F, Dai B, Zhang W, et al. HLA-A, HLA-B, HLA-DRB1 allele and haplotype frequencies in 6384 umbilical cord blood units and transplantation matching and engraftment statistics in the Zhejiang cord blood bank of China. Int J Immunogenet 2014;41:13–9. doi:10.1111/iji.12064.

[45] He D, Wang J, Yi L, Guo X, Guo S, Guo G, et al. Association of the HLA-DRB1 with Scleroderma in Chinese Population. PLoS One 2014;9:e106939. doi:10.1371/journal.pone.0106939.

[46] Li X-F, Zhang X, Chen Y, Zhang K-L, Liu X-J, Li J-P. An Analysis of HLA-A, -B, and -DRB1 Allele and Haplotype Frequencies of 21,918 Residents Living in Liaoning, China. PLoS One 2014;9:e93082. doi:10.1371/journal.pone.0093082.

[47] Zhao J-J, Wang X-B, Luan Y, Liu J-L, Liu L, Jia H-Y. Association of human leukocyte antigen gene polymorphism and mesangial proliferative glomerulonephritis in a large population-based study. Biomed Reports 2013;1:751–6. doi:10.3892/br.2013.152.

[48] Liu B, Xiong L, Tian C, Zhou Q, Zhong Y, Li A, et al. HLA-DRB1*12:02:01 plays a protective role against coronary artery disease in women of southern Han Chinese descent. Hum Immunol 2012;73:122–6. doi:10.1016/j.humimm.2011.11.003.

[49] He J, Li Y, Bao X, Qiu Q, Yuan X, Xu C, et al. Common and well-documented (CWD) alleles of human leukocyte antigen-A, -B, -C, -DRB1, and -DQB1 loci for the Chinese Han population do not quite correlate with the ASHI CWD alleles. HIM 2012;73:61–6. doi:10.1016/j.humimm.2011.06.005.

[50] Li X, Liu W, Wang H, Jin X, Fang S, Shi Y, et al. The influence of HLA alleles and HBV subgenotyes on the outcomes of HBV infections in Northeast China. Virus Res 2012;163:328–33. doi:10.1016/j.virusres.2011.10.020.

[51] Sun C, Wei L, Luo F, Li Y, Li J, Zhu F, et al. HLA-DRB1 alleles are associated with the susceptibility to sporadic Parkinson’s disease in Chinese Han population. PLoS One 2012;7:e48594. doi:10.1371/journal.pone.0048594.

[52] Qin Qin P, Su F, Xiao Yan W, Xing Z, Meng P, Chengya W, et al. Distribution of human leucocyte antigen-A, -B and -DR alleles and haplotypes at high resolution in the population from Jiangsu province of China. Int J Immunogenet 2011;38:475–81. doi:10.1111/j.1744-313X.2011.01029.x.

[53] Wang H, Dai Y, Qiu W, Zhong X, Wu A, Wang Y, et al. HLA-DPB1*0501 is associated with susceptibility to anti-aquaporin-4 antibodies positive neuromyelitis optica in Southern Han Chinese. J Neuroimmunol 2011;233:181–4. doi:10.1016/J.JNEUROIM.2010.11.004.

[54] Jiang H-W, Tian H-Q, Liu H, Li N, Zhao Y, Zhang F-R. Association of the HLA-DRB1 locus with syphilis in a Chinese population. Int J Infect Dis 2011;15:e342-5. doi:10.1016/j.ijid.2011.01.008.

[55] Xie Y-C, Qu Y, Sun L, Li H-F, Zhang H, Shi H-J, et al. Association between HLA-DRB1 and myasthenia gravis in a northern Han Chinese population. J Clin Neurosci 2011;18:1524–7. doi:10.1016/j.jocn.2011.05.002.

[56] Lu W-S, Zhang W-Y, Li Y, Wang Z-X, Zuo X-B, Cai L-Q, et al. Association of HLA-DRB1 alleles with keloids in Chinese Han individuals. Tissue Antigens 2010;76:276–81. doi:10.1111/j.1399-0039.2010.01509.x.

[57] Shen C, Zhu B, Ye S, Liu M, Yang G, Liu S, et al. Allelic diversity and haplotype structure of HLA loci in the Chinese Han population living in the Guanzhong region of the Shaanxi province. Hum Immunol 2010;71:627–33. doi:10.1016/j.humimm.2010.02.012.

[58] Wu X-M, Wang C, Zhang K-N, Lin A-Y, Kira J-I, Hu G-Z, et al. Association of susceptibility to multiple sclerosis in Southern Han Chinese with HLA-DRB1, -DPB1 alleles and DRB1- DPB1 haplotypes: distinct from other populations. Mult Scler 2009;15:1422–30. doi:10.1177/1352458509345905.

[59] Hei A-L, Li W, Deng Z-H, He J, Jin W-M, Du D, et al. Analysis of high-resolution HLA-A, -B, -Cw, -DRB1, and -DQB1 alleles and haplotypes in 718 Chinese marrow donors based on donor-recipient confirmatory typings. Int J Immunogenet 2009;36:275–82. doi:10.1111/j.1744-313X.2009.00866.x.

[60] Zhang F, Liu H, Chen S, Wang C, Zhu C, Zhang L, et al. Evidence for an association of HLA-DRB1*15 and DRB1*09 with leprosy and the impact of DRB1*09 on disease onset in a Chinese Han population. BMC Med Genet 2009;10:133. doi:10.1186/1471-2350-10-133.

[61] Huang X, Ling H, Mao W, Ding X, Zhou Q, Han M, et al. Association of HLA-A, B, DRB1 alleles and haplotypes with HIV-1 infection in Chongqing, China. BMC Infect Dis 2009;9:201. doi:10.1186/1471-2334-9-201.

[62] Paximadis M, Mathebula TY, Gentle NL, Vardas E, Colvin M, Gray CM, et al. Human leukocyte antigen class I (A, B, C) and II (DRB1) diversity in the black and Caucasian South African population. Hum Immunol 2012;73:80–92. doi:10.1016/j.humimm.2011.10.013.

[63] Lombard Z, Dalton D-L, Venter PA, Williams RC, Bornman L. Association of HLA-DR, -DQ, and Vitamin D Receptor Alleles and Haplotypes with Tuberculosis in the Venda of South Africa. Hum Immunol 2006;67:643–54. doi:10.1016/j.humimm.2006.04.008.

[64] Bhimma R, Hammond MG, Coovadia HM, Adhikari M, Connolly CA. HLA class I and II in black children with hepatitis B virus-associated membranous nephropathy. Kidney Int 2002;61:1510–5. doi:10.1046/j.1523-1755.2002.00287.x.

[65] Patel M, Wadee AA, Galpin J, Gavalakis C, Fourie AM, Kuschke RH, et al. HLA class I and class II antigens associated with multiple myeloma in southern Africa. Clin Lab Haematol 2002;24:215–9. doi:10.1046/j.1365-2257.2002.00448.x.

[66] Pirie FJ, Hammond MG, Motala AA, Omar MAK. HLA class II antigens in South African Blacks with type I diabetes. Tissue Antigens 2001;57:348–52. doi:10.1034/j.1399-0039.2001.057004348.x.

[67] Schipper RF, D'Amaro J, Bakker JT, Bakker J, van Rood JJ, Oudshoorn M. HLA gene haplotype frequencies in bone marrow donors worldwide registries. Hum Immunol 1997;52:54–71.
